# Factors predicting long-term weight maintenance in anorexia nervosa: a systematic review

**DOI:** 10.1007/s40519-024-01649-5

**Published:** 2024-04-06

**Authors:** Lydia Maurel, Molly MacKean, J. Hubert Lacey

**Affiliations:** 1 Schoen Clinic Chelsea, London, UK; 2 Schoen, Birmingham, UK; 3 St George’s, University of London, London, UK

**Keywords:** Eating disorders, Anorexia nervosa, Weight maintenance, Recovery, Weight restoration

## Abstract

**Purpose:**

Eating disorder recovery is a poorly defined concept, with large variations among researchers’ definitions. Weight maintenance is a key aspect of recovery that remains relatively underexplored in the literature. Understanding the role of weight maintenance may help guide the development of treatments. This paper aims to address this by (1) investigating the factors predicting long-term weight maintenance in anorexia nervosa (AN) patients; (2) exploring differences in predictive factors between adolescent and adult populations; and (3) exploring how weight maintenance is conceptualised in the literature. Methods: We conducted a systematic review following PRISMA guidelines to address our research questions. Five databases were searched and filtered according to our exclusion criteria.

**Results:**

From the search, 1059 studies were yielded, and 13 studies were included for review. A range of weight, biological and psychological factors were found to predict weight maintenance among these papers. BMI at admission and discharge from inpatient treatment was the most common predictor among the papers. Few studies investigated biological factors and mixed evidence was found for psychological factors. We found no observable differences between adult and adolescent populations. Finally, weight maintenance was defined and measured differently across studies.

**Conclusion:**

This review’s findings can help contribute to a well-rounded understanding of weight maintenance, and ultimately, of recovery. This can help support clinicians in tailoring interventions to improve long-term outcomes in AN. Future research should aim to replicate studies to better understand the relationship between the factors identified and weight maintenance.

**Level I:**

Systematic review.

## Introduction

Eating disorders are severe mental health conditions that negatively impact an individual’s physical, psychological and social functioning [1]. The prevalence and severity of eating disorder presentations have increased significantly over the last few years, with hospital admissions in the UK increasing by 84% in the last five years [2]. The recent COVID-19 pandemic further contributed, in part, to this increase, whereby individuals with eating disorders faced significant challenges such as increased social isolation, a reduced sense of control, and limited access to healthcare services [3, 4]. Taken together, these pressures have meant that eating disorder services have struggled to meet demand and healthcare providers face the ongoing need to develop and adapt treatments accordingly.

Clinicians have highlighted concerns around long-term outcomes for patients following eating disorder treatment, in particular relapse. For example, relapse rates of 31% have been reported in anorexia nervosa (AN) [5], highlighting the importance of understanding contributing factors. Studies have explored possible mechanisms behind AN relapse and have found a wide range of possible factors. Frostad et al. [6] found BMI at discharge was a significant predictor of relapse in adults and adolescents. This lies in contrast to other studies that have found factors such as weight and shape concerns [5] having the binge–purge subtype of AN, having more motivation to recover at different points in treatment, and the severity of pre-treatment checking behaviour [7] to be significant predictors of relapse. Whilst these findings may support the adaptations of future treatments, a drawback of focusing on relapse is the heavy emphasis on preventing negative outcomes, rather than promoting positive change. These are two separate facets of long-term AN outcomes, and a substantial focus on preventing relapse may disempower an individual in their journey.

The promotion of positive outcomes in AN can be viewed through a recovery-focused lens. Numerous factors have been identified as predictors of recovery or positive outcomes, including personality traits [8, 9], family relations [10], impulsivity [9], selflessness [11], and self-esteem [12, 13]. As the aim for patients, families and clinicians is full recovery from AN, this has led to a comprehensive literature base on factors impacting AN recovery, and subsequently, a vast landscape of possible definitions of recovery [14]. Many researchers have attempted to operationalise ‘recovery’, with a widely accepted modern view that this should include a combination of biological, physical, and cognitive constructs [15], as well as measures of psychological and social wellbeing [16]. However, the concept of recovery remains somewhat abstract due to the variability in the individual’s experience and the personal nature of recovery for each person, which together have led to difficulties with measuring recovery, its predictors and with producing replicable studies [8].

An important aspect of recovery is weight maintenance, which refers to the sustained management of weight within a healthy range over time. Underweight individuals with AN have a twofold challenge when it comes to weight: weight gain and weight maintenance. Research has investigated factors that contribute to weight gain in various clinical settings [17, 18]. Byrne et al. [17] found that parental self-efficacy was a significant predictor of weight gain for adolescents undergoing family-based treatment for AN. Nyman-Carlsson et al. [18] investigated pre-treatment factors predicting weight gain in a sample of young adult women and found that different predictors were significant depending on the type of treatment received. These factors included levels of emotion dysregulation and deficits in one’s ability to understand and cope with emotions. Research has also demonstrated early weight gain during treatment is a strong predictor of overall weight gain, as well as full recovery [19, 20]. Whilst studies have investigated weight gain, there is little research on factors that impact weight maintenance. This is surprising given weight maintenance is a primary aim of AN treatments. Furthermore, research has found that weight maintenance is an essential part of full recovery outcomes [21, 22]; for example, Rigaud et al. [22] found in a sample of adult inpatients with AN that more years spent relapse-free increased the probability of reaching full recovery with each year.

Better understanding the factors that impact weight maintenance can provide a focus on the positive aspects of AN trajectories and may support services to sustain existing improvement, including maximising current successful aspects of treatment. Furthermore, this perspective would allow us to focus on weight as an important aspect of positive change, whilst acknowledging that there are other relevant factors within recovery. This specific focus prevents researchers from becoming lost in an abstract world of ‘recovery’. In this context, recovery lacks a clear conceptualisation due to the wide number of relevant factors and its personal nature. Investigating weight maintenance in more depth can contribute to establishing a better understanding of recovery, and at the same time, the specificity of weight maintenance may allow findings to be more effectively applied in clinical settings.

This study reviews the current literature on factors associated with long-term weight maintenance in AN specifically, rather than eating disorders as a whole, given the heterogeneity of eating disorders and the likelihood of different factors affecting weight maintenance. Long-term weight maintenance is considered as defined by the papers included in the review and its definition will be further discussed in this paper.

This study further aims to investigate whether any identified factors vary between adult and adolescent populations. AN impacts adolescents and adults differently due to differences across developmental stages; for example, younger patients may have poorer medical outcomes as compared with adults [23]. Furthermore, therapeutic approaches differ according to age [24]. It is therefore possible that the factors maintaining long-term weight maintenance may also differ for each population. Another important reason to investigate differences between adults and adolescents is their differences in treatment outcome. Despite poorer morbidity, research suggests that adolescents have overall better outcomes after treatment for their eating disorder, and that the effect of factors such as early weight gain is larger for children than for adults [25, 26]. This suggests that it is important to investigate patterns in factors that predict aspects of recovery, such as weight maintenance, as this may help explain overall differences in recovery rates for each group.

This paper aims to answer the following questions:

1. What are the factors associated with long-term weight maintenance following weight restoration in AN?
2. What are the similarities and differences in the factors associated with long-term weight maintenance between adult and adolescent AN populations?
3. How is long-term weight maintenance conceptualised in AN literature?

## Methods

### Search strategy

A systematic search of the literature was conducted by one reviewer, following PRISMA guidelines, between February and August 2022 using PubMed, MEDLINE, PsycINFO, Cochrane Library and Wiley Online Library. A pre-defined list of search terms was used to generate the literature search, including a combination of: “eating disorders”; “long term weight restoration”; “long term weight maintenance”; “anorexia nervosa”; “weight maintenance” and “restrictive eating disorder”.

### Eligibility criteria and selection process

Due to the lack of available studies in this research area, no limits were placed on the patient demographics, type of intervention or study design. Studies were excluded using the following primary criteria:

- Measuring weight maintenance after weight loss, in obesity and binge eating populations;
- Examining factors that predict poor outcomes;
- Examining factors that predict global outcomes, including psychological improvement; and
- Measuring factors that predict long-term improvements in weight as a continuous variable.

Inclusion criteria included:

- Studies measuring factors associated with and/or predicting weight maintenance in AN populations; and
- English language studies.

For the purpose of this paper, studies met the condition of weight maintenance if they identified a weight or weight range to be sustained over a period of time. No limits were placed on the defined length of time required for a participant’s weight to be considered maintained, and patterns in these definitions will be discussed in the results.

**Fig. 1:**
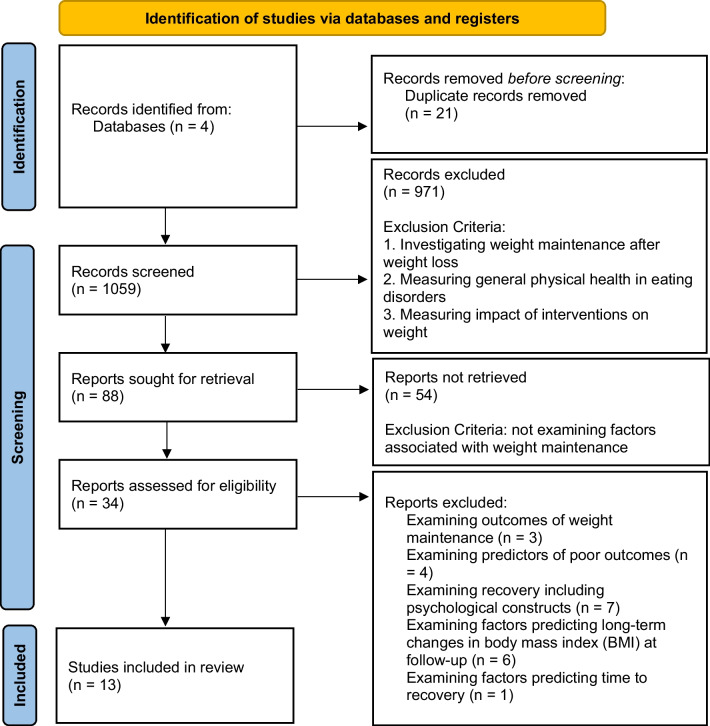
PRISMA 2020 flow diagram

The reviewer screened abstracts and retrieved full-texts of appropriate studies using the eligibility criteria above. Reference lists of reports that were assessed for eligibility were also searched for any appropriate studies. Reference lists were searched at this point in the retrieval process as the reports retrieved thus far were likely to be the most appropriate and may refer to other studies that contribute to their reports on weight maintenance. Figure 1 outlines the selection process for the studies included in this review, including full exclusion criteria. The third author was consulted regarding any studies that required further consultation to determine if they met inclusion or exclusion criteria.

A data extraction sheet was used by two reviewers to independently gather data on study purpose and design, intervention details, participant characteristics, definition of weight maintenance, any other measures and results/outcomes were sought from the retrieved full-text studies. These studies were then examined by the third author for clarification on study details and outcomes where needed.

The quality of the included studies was evaluated using the Quality Assessment Tool (QAT) [25].

## Results

The initial search yielded 1059 studies which were then screened for eligibility. Eighty-eight studies remained, and their abstracts were reviewed, with any papers that did not investigate factors associated with weight maintenance in AN being excluded at this stage. Thirteen studies remained, and the full texts were retrieved and assessed for eligibility. In addition, the reference lists of these 13 studies were reviewed, alongside any papers that had cited them, yielding a total of 21 additional studies. Combined, this resulted in the retrieval of full-texts for 34 studies. Twenty-one studies were excluded because they met the exclusion criteria, resulting in 13 studies for review in this paper. The process of selecting studies for review, following PRISMA guidelines, is depicted in Fig. 1.

Sample characteristics of all included studies, as well as results from the quality assessment, are presented in Table 1. All studies examined factors that influence long-term weight maintenance in individuals with AN as part of their research, although some studies did not investigate this as a primary aim. All the included studies were published between 2007 – 2021. In all 13 studies, the samples consisted of patients who had been admitted to inpatient or day patient programmes [26–38] and in 6 of these studies, patients had been discharged to outpatient programmes [26–30, 38].

**Table 1:** Summary of included studies, including sample characteristics, results and quality assessment

Authors	Countries represented	N	Mean age in years (adults/adolescents)	Design	Definition of weight maintenance	Weight Maintenance Rate (%)	Factors Investigated in relation to weight maintenance	Follow-up duration (months or years)	Results summary	Quality assessment
El Ghoch et al. [26]	Italy	54	25.6 (adults)	Longitudinal Prospective Study Stepped down from inpatient to day patient to outpatient treatment Adapted version of enhanced cognitive-behavioural therapy (CBT) for eating disorders (CBT-E)	BMI ⩾ 18.5 kg/m^2^	48.15%	Total body fat percentage (%) Trunk fat percentage (%) Body Mass Index (BMI) at discharge (kg/m^2^)	12 months	BMI at inpatient discharge significantly predicted weight maintenance at follow-up Total body fat and trunk fat at inpatient discharge did not significantly predict weight maintenance at follow-up	Good
Uniacke et al. [27]	United States Canada	93	23.3 (adults and adolescents)	Longitudinal prospective study Inpatient or day patient discharged to outpatient treatment CBT-E Secondary data from Kaplan et al. [30]	BMI ⩾ 18.5 kg/m^2^	43%	Weight suppression BMI (kg/m^2^)	6 and 12 months	Weight suppression and its interaction with BMI did not significantly predict weight maintenance at follow-up	Fair
Kim et al. [28]	United States	41	25.0 (adults)	Longitudinal prospective study Multi-site Inpatient treatment Pooled cohort from Bodell & Mayer [38]; Mayer et al. [39]	BMI ⩾ 18.5 kg/m^2^ Required for 8 weeks before follow-up	48.8%	Regression model with age and BMI: Body fat percentage (%) Log(leptin) Log(leptin)_fat adj_	12 months	Higher log-leptin and percent body fat, as well as fat-adjusted leptin, independently predicted weight maintenance at 1-year follow-up	Good
Castro-Fornieles et al. [29]	Spain	40	14.4 (adolescents)	Longitudinal prospective study Inpatient programme stepped down to outpatient treatment	BMI ⩾ 18.5 kg/m^2^	62.5%	Readiness to recover (Anorexia Nervosa Stages of Change Questionnaire—ANSOCQ) BMI at admission (kg/m^2^)	9 months	High motivation to change at discharge and a higher BMI at admission predicted weight maintenance at follow-up	Good
Kaplan et al. [30]	United States Canada	93	23.3 (adults and adolescents)	Longitudinal prospective study using data from an RCT Patients were randomly assigned to fluoxetine or placebo condition following minimal weight restoration Patients were offered CBT for AN relapse prevention Inpatient or day patient discharged to outpatient treatment	BMI ⩾ 18.5 kg/m^2^ Definition also required participants to remain in treatment and not exhibit severe medical or psychological decline	43%	Socio-economic status code (Hollingshead Occupational Categories) Subtype (binge/purge or restricting) Duration of AN (years) Number of previous hospitalisations Number of Axis I SCID diagnoses Past suicide attempts BMI at admission (kg/m^2^) Weight change over first 28 days of maintenance (lb/week) Patient estimate of chance of weight maintenance (0–100%) Patient estimate of chance of relapse (0–100%) Specific eating disorder psychopathology measures using: Eating Disorder Examination, 12th Edition (EDE) for: EDE shape concerns and EDE weight concerns Mizes Anorectic Cognitions (MAC) scale Yale–Brown–Cornell Eating Disorder Scale (YBC EDS) Exercise during last month of initial treatment Commitment to Exercise Scale (CES) Eating Disorder Inventory (EDI)—EDI Ineffectiveness Scale	6 and 12 months	Higher BMI at discharge, as well as a lower rate of weight loss immediately following discharge, predicted weight maintenance at 6 and 12 months	Fair
Glasofer et al. [31]	United States	194	26.02 (adults and adolescents)	Longitudinal prospective study Inpatient treatment Behavioural approach	Category 1: BMI ≥ 18.5 kg/m^2^ Category 2: BMI ≥ 19.5 kg/m^2^	45.8% 32.1%	Duration of illness (years) Becks Anxiety Inventory (BAI) Becks Depression Inventory (BDI) Spielberger State Trait Anxiety Inventory-Trait version (STAI-T) Eating Disorder Examination-Questionnaire (EDE-Q) global AN subtype, binge-purge Admission BMI (kg/m^2^) Discharge BMI (kg/m^2^) Number of annual assessments completed	60 months	Higher BMI at discharge was significantly associated with weight maintenance at follow-up Higher BMI at admission was also associated with weight maintenance at follow-up, although the effect was less pronounced	Good
Boehm et al. [32]	Germany	76	15.84 (adolescents)	Longitudinal prospective study Inpatient treatment comprising behavioural, cognitive-behavioural and body-oriented psychotherapeutic elements	BMI ≥ 18.5 kg/m^2^	55.8% = ’good’ outcome	BPI quotient (measuring perceptual body image distortion) Body mass index standard deviation score (BMI-SDS) increase within the first 28 days (early weight gain) BMI-SDS increase until discharge Subtype of AN Medication intake (antidepressants and neuroleptics) Duration of follow-up	Mean = 3.7 years (range = 1–7.5)	None of the predictor variables significantly predicted the physical outcome alone, which included having a BMI above 18.5 kg/m^2^ and mensus at follow-up	Good
Calugi et al. [33]	Italy	66	26.1 (adult)	Longitudinal prospective study Inpatient treatment CBT-E based (adapted for inpatients)	BMI ⩾ 18.5 kg/m^2^	Details Unclear	Admission and discharge measures of components of body-image concerns: ‘preoccupation with shape or weight’ ‘fear of weight gain’ ‘feeling fat’ The distinct components of body image concerns were measured using single items from the Italian validated version of the EDE12.0	6 and 12 months	Admission and discharge measures of all 3 components of body image concerns were related to higher odds of reaching weight maintenance at 6-month follow-up ‘fear of weight gain’ was associated with a higher likelihood of reaching weight maintenance at 12-month follow-up (However, no p-values are reported)	Good
Forman et al. [34]	United States	700	15.3 (adults and adolescents)	Retrospective chart review Inpatient, day patient and outpatient treatment at different sites	> 90%mBMI	Details Unclear	Age (Years) Gender (Male/Female) Race (White/Not White) %MBMI at intake Duration of illness (months) more or less than 18 months Diagnosis (AN, atypical AN, ARFID) Higher level of care before intake (Yes/No)	12 months	The only significant predictor of weight recovery was %MBMI at intake when controlling for age, gender, illness duration, diagnosis and level of care	Good
Brewerton and Costin [35]	United States	118 (AN = 66)	AN = 31.4 (adults)	Retrospective analysis of questionnaires from respondents receiving inpatient treatment at admission and follow-up post-treatment	Weight recovery = BMI > 18	AN = 70% (BMI ⩾ 18) 42% had a good outcome (BMI > 18 AND resumption of normal menses)	Age (years) Illness duration (years) Age of onset (years) Number of previous hospitalisations Length of stay (days) Admission BMI (kg/m^2^) Discharge BMI (kg/m^2^) Admission Becks Depression Inventory (BDI) Discharge BDI Admission EDI-2 subscale scores Discharge EDI-2 subscale scores Admission frequencies of eating disordered behaviours Discharge frequencies of eating disordered behaviours	AN = 4.6 years	Discharge BMI was the only significant predictor of weight maintenance at follow-up	Fair
Cooper et al. [36]	United States	146	30.07 (adults and adolescents)	Longitudinal prospective study Integrated inpatient-partial hospitalisation programme Structured behavioural modification	BMI ⩾ 19 kg/m^2^	59.60%	Change scores for normative eating self-efficacy Body image self-efficacy Drive for thinness Body dissatisfaction	6 months	Discharge BMI, normative eating self-efficacy at admission, change in normative eating self-efficacy, and normalised eating behaviours were all associated with weight restoration at follow-up	Good
Redgrave et al. [37]	United States	99	32.55 (adults)	Longitudinal prospective study Integrated step-down inpatient-partial hospitalisation programme Structured behavioural modification	BMI ⩾ 19 kg/m^2^	Long-term ill = 58.73% Short-term ill = 52.78% Mean = 55.76%	Admission BMI (kg/m^2^) Discharge BMI (kg/m^2^) Lifetime nadir BMI (kg/m^2^) Illness duration	6 months	Only discharge BMI significantly predicted weight maintenance at 6-month follow-up	Good
Bodell and Mayer [38]	United States	21	Full, Good or Fair Outcome = 27.9 Poor Outcome = 25.4 (adults)	Longitudinal prospective study Inpatient treatment	“Good outcome” = BMI ⩾ 18.5 kg/m^2^; normal menses, may have some behavioural/psychological symptoms “Fair outcome”—BMI ≥ 18.5 kg/m^2^; amenorrhea	52.4% = Full, Good or Fair outcome	Percent body fat at discharge from inpatient treatment (%)	12 months	The relationship between body fat percentage and clinical outcome did not reach significance	Good

The mean age of participants across all studies ranged from 14.40 to 32.55 years. Data from studies show that the average weight maintenance rate for the participants ranged from 32.10% to 62.50%. Six studies included only adults [26, 28, 33, 35, 37, 38], two studies included only adolescents [29, 32], and five studies included both adults and adolescents [27, 30, 31, 34, 36]. The combined sample size across all the studies was 1689. Significant findings and p-values from the included papers are presented in Table 2.

**Table 2:** Factors associated with/predicting weight maintenance

Authors	Variable	Measure moment (month or years)	*P*
El Ghoch et al. [26]	Discharge BMI (from inpatient care)	12 months	0.002
Kim et al. [28]	Higher log leptin pre-discharge (from inpatient care)	12 months	0.021
Kim et al. [28]	Higher leptin levels (fat-adjusted) pre-discharge (from inpatient care)	12 months	0.029
Kim et al. [28]	Body fat percentage pre-discharge (from inpatient care)	12 months	0.010
Castro-Fornieles et al. [29]	Readiness to recover at discharge (from inpatient care)	9 months	0.003
Castro-Fornieles et al. [29]	Admission BMI (to inpatient care)	9 months	0.008
Kaplan et al. [30]	Discharge BMI (from inpatient care)	6 months	0.001
Kaplan et al. [30]	Discharge BMI (from inpatient care)	12 months	0.003
Kaplan et al. [30]	Rate of weight loss in first 28 days of outpatient treatment post-discharge from inpatient care	6 months	0.000
Kaplan et al. [30]	Rate of weight loss in first 28 days of outpatient care	12 months	0.000
Glasofer et al. [31]	Discharge BMI (from inpatient care)	60 months	Good outcome category 1 = 0.012 Good outcome category 2 = 0.001
Glasofer et al. [31]	Admission BMI (to inpatient care)	60 months	Good outcome category 1 = 0.002
Forman et al. [34]	Baseline %MBMI (inpatient, day patient and outpatient care)	12 months	0.0004
Brewerton and Costin [35]	Discharge BMI	4.6 years (average)	0.015
Cooper et al. [36]	Weight restoration at discharge	6 months	< 0.001
Cooper et al. [36]	Normative eating self-efficacy at admission	6 months	0.023
Cooper et al. [36]	Change in normative eating self-efficacy	6 months	0.002
Redgrave et al. [37]	Discharge BMI (from inpatient care/partial hospitalisation)	6 months	0.012

### Definition of weight maintenance

There was a range of weight maintenance definitions across the studies, with different definitions for both adult and adolescent samples. Eleven studies used a measure of between BMI ≥18 and 19.5 [26–33, 35–37], whilst Forman [34] used > 85% median BMI (%mBMI).

All studies took measures at three or more time points: admission, discharge and one or more follow-up points. Follow-up ranged from 6 months to 5 years. Six studies provided measures of weight between discharge and follow-up to ensure weight maintenance was sustained during the given time period [27, 28, 30, 31, 35, 38]. From these, two studies used only in-person measures [27, 30], two studies used only online/phone measures [31, 35], and two studies used a combination of both [28, 38]. Furthermore, three studies defined the time in weight maintenance needed for a patient to be considered in ‘maintenance’, specifying a requirement ranging from 4 to 8 consecutive weeks [27, 28, 30].

### Themes

After reviewing the included articles, our findings can be grouped into three themes: BMI/weight variables; biological markers; and psychological markers.

### Weight variables—BMI

Overall, BMI was most commonly investigated as a predictor of weight maintenance across the studies [26, 29–32, 35, 37].

The most common finding was that BMI at discharge from inpatient treatment significantly predicted weight maintenance at follow-up [26, 30, 31, 35, 37]. Kaplan [30] found that women with a higher BMI at discharge from intensive treatment (inpatient or day patient) were more likely to maintain their weight at 6- and 12-month follow-up. El Ghoch [26] found discharge BMI significantly predicted weight maintenance at 12-month follow-up in a sample of inpatient women. Redgrave [37] also found similar results using a more stringent measure of maintenance (BMI ≥ 19 kg/m^2^) at 6-month follow-up, and two studies found similar findings at longer term follow-up, namely up to 5 years [31, 35]. These five studies reported that participants had a discharge BMI between 19.0 ± 3.3 kg/m^2^ and 20.3 ± 0.5 kg/m^2^.

Alternatively, only two studies found that a higher BMI at admission to inpatient treatment significantly predicted weight maintenance up to 1-year follow-up [29, 31]. Castro-Fornieles [29] found that admission BMI predicted weight maintenance at 9-month follow-up. Glasofer [31] found that BMI at admission predicted weight maintenance at 18.5 kg/m^2^, but not at a more stringent cut off at 19.5 kg/m^2^, whereas discharge BMI predicted maintenance in both maintenance at 18.5 kg/m^2^ and at 19.5 kg/m^2^. These studies reported a BMI at admission between 15.5 ± 1.4 kg/m^2^ and 16.0 ± 1.86 kg/m^2^.

However, not all studies found BMI to be a significant predictor. Boehm [32] used a different measure of BMI and found that increases in BMI standard deviation scores did not significantly predict weight maintenance at follow-up, which was a mean of 3.7 years after the start of inpatient treatment.

### Other weight variables

Some studies investigated the predictive value of other weight-related variables on long-term weight maintenance. Forman [34] found that %MBMI was a significant predictor of weight maintenance at 1-year follow-up in a sample of adolescents and young adults, such that for each 5% increase in baseline %MBMI, patients were 1.69 times more likely to reach weight maintenance.

Uniacke [27] investigated the impact of weight suppression using data from Kaplan’s [30] study. Weight suppression refers to the difference between a person’s previous highest weight and their current weight [40]. Whilst previous research has found that weight suppression predicts weight gain outcomes [40], Uniacke [27] found neither weight suppression, nor the interaction between weight suppression and BMI (measured at start of outpatient treatment), significantly predicted weight maintenance at follow-up.

One study investigated the predictive value of early weight gain on long-term outcomes. Kaplan [30] found that the rate of weight change, namely a lower rate of weekly weight loss, in the first 28 days of outpatient CBT treatment was a significant predictor of weight maintenance.

### Biological markers

Three studies investigated the impact of biological factors related to weight [26, 28, 38]. Two studies found that body fat percentage, measured using a whole-body DXA scan and MRI imaging, did not significantly predict weight maintenance at 12-month follow-up [26, 38]. However, Kim [28] found that both body fat percentage and higher levels of leptin (fat-adjusted) pre-discharge from inpatient treatment, measured using whole-body MRI imaging, significantly predicted weight maintenance at 12-month follow-up.

### Psychological markers

The identified studies in this paper investigated a range of psychological markers with mixed findings. Some studies found significant findings for predictors related to motivation and belief in oneself to change [29, 36]. Castro-Fornieles [29] found that readiness to recover significantly predicted weight maintenance at 9-month follow-up in a sample of adolescents. Cooper [36] found that normative eating self-efficacy at admission was significantly associated with long-term weight maintenance, with participants 4.65 times more likely to have maintained weight at 6-month follow-up for each one-unit increase in normative eating self-efficacy scores from admission to follow-up.

Three studies investigated the impact of body image concern components on weight maintenance outcomes namely: ‘fear of weight gain’, ‘preoccupation with weight or shape’, ‘feeling fat’, body image distortion, and body image self-efficacy [32, 33, 36]. Calugi [33] explored a range of variables, measured by using items taken from the Eating Disorder Examination 12.0D at the end of treatment, and found that lower scores for ‘fear of weight gain’ at baseline were associated with a higher likelihood of maintaining weight at 6- and 12-month follow-up in a sample of young women. Calugi [33] also found that lower scores of ‘preoccupation with shape or weight’, and ‘feeling fat’ predicted weight maintenance at 6-month follow-up, but not at 12-month follow-up. However, the authors did not include significance values in their findings therefore it is unclear whether these measures are significant predictors. In contrast, Boehm [32] investigated a separate facet of body image, specifically perceptual body image distortion, referring to the accuracy of comparison between one’s perceived and actual body size [41], and found this did not significantly predict weight maintenance, although it was a significant predictor of long-term global outcome (including psychological outcomes). In line with this, Cooper [36] found that improvements in body dissatisfaction and body image self-efficacy from admission to follow-up, namely the belief in oneself to complete everyday tasks without being held back by body image concerns [42], did not significantly predict long-term weight maintenance either.

Other studies found that psychological variables, including anxiety symptoms, depression symptoms, eating disorder psychopathology, expectations for recovery, personality traits and quality of life did not significantly predict long-term weight maintenance [30, 35, 36].

### Difference between adult and adolescent samples

The studies were reviewed to examine whether any predictors differentiated the adult and adolescent patient groups. None of the studies with both adolescent and adult samples analysed differences in predictors between these two groups. Studies looking at biological markers used only adult samples. Otherwise, there were no observable patterns in the data to suggest that any variable has been found to predict weight maintenance more consistently in adult or adolescent samples.

## Discussion

The overarching aim of this review was to explore the factors predicting long-term weight maintenance in adults and adolescents with AN, and then compare any differences between adults and adolescents. Another aim was to evaluate how weight maintenance is defined and measured among these papers. A literature review was conducted following the PRISMA framework, resulting in 13 studies. The review identified a range of weight, biological and psychological factors investigated in relation to weight maintenance, but also found that the concept of weight maintenance varied among the studies.

### BMI at admission and discharge

The most common significant finding across studies was that BMI at admission and discharge from inpatient treatment significantly predicted weight maintenance across both adult and adolescent samples.

Our finding that admission BMI predicts weight maintenance is mirrored in the literature on recovery, as admission BMI has been found to significantly predict treatment outcome and recovery [43, 44]. However, we found that BMI at discharge from treatment was a more common significant predictor of weight maintenance than admission BMI among the included studies. This finding is important as there is little research on the predictive value of discharge BMI in the recovery and relapse literature. A possible reason for this finding is that patients with a higher discharge BMI would have a wider margin for some weight loss to remain in the ‘maintenance’ category as compared to those with a lower discharge BMI, making maintenance easier from a weight perspective. It is also possible that higher BMI at discharge correlates with increased cognitive function recovery, which is linked to increased cognitive flexibility [45]. This may support individuals in their efforts to maintain their weight after treatment, although further research is needed to investigate these relationships.

Research on recovery has investigated changes in BMI during inpatient treatment, rather than BMI before or after treatment, and found that larger changes in BMI between admission and discharge were a significant predictor of remission at follow-up, whereas admission BMI was not a significant predictor [46]. It would be interesting to investigate this construct further in relation to long-term weight maintenance, in order to better understand the predictive value and relationships between admission BMI, discharge BMI and weight gain during treatment.

Some papers included in this review investigated the predictive value of other weight-related variables, such as weight suppression and rate of weight change in treatment [27, 28]. Whilst some findings are significant, given that there has been little replication of any significant findings on these weight variables, future research is needed.

### Biological factors

This review also identified studies that investigated biological factors predicting weight maintenance, namely body fat and leptin levels. Whilst studies investigating body fat found this was not a significant predictor of weight maintenance [26, 38], one study found that body fat percentage and leptin levels at discharge significantly predicted weight maintenance at follow-up [28]. This may correlate with our finding that discharge BMI predicts weight maintenance, as identified in this review. Given the relationship between body fat percentage and leptin levels with a person’s weight for height, these findings may be linked [6].

### Psychological factors

The present study found a range of psychological factors affecting weight maintenance among the literature. Some studies found that factors related to self-efficacy and motivation to change significantly predicted weight maintenance [36, 39]. Research suggests that increased self-efficacy predicts end-of-treatment outcomes in eating disorder populations [12, 47], and it is possible that this may help to support individuals with AN in maintaining their weight after treatment, helping them cope with difficulties and challenges they may face during this process [48]. However, the other studies looking at body image constructs in this review found mixed results [32, 33, 36]. Research suggests that psychological factors tend to take longer than physical factors to improve [49], and body image disturbance is suggested to shift in the later stages of recovery [12]. This may explain these inconsistent findings in the present review, though this should be interpreted with caution.

### Adult vs adolescent samples

Our second aim was to explore whether there were any differences between adult and adolescent samples in factors that predict weight maintenance. We found no observable patterns in results from the included studies between age groups. This may be due to the limited number of studies in this review, which will have impacted our ability to observe patterns; it would be important for future research to investigate this further. An understanding of the role of different factors in weight maintenance may help clinicians to tailor interventions according to age group, as adolescents and adults face different challenges when overcoming eating disorders. [50].

### Conceptualisation of weight maintenance

We found that many studies used different definitions of weight maintenance, including different weight cut-offs and time periods required for weight restoration to be considered maintenance. This makes it difficult to compare findings across studies because different factors may have varying predictive value. For example, one study using a different measure of BMI, namely BMI-SDS, found this did not predict weight maintenance [32], which may suggest that the measurements used may impact findings. Further, one study with a notably longer-term follow-up period [32] had non-significant findings regarding a range of variables. This highlights the need for more consistent measurements and follow-up periods, to gain a better understanding of predictive variables in weight maintenance.

Despite this, many studies used the weight criterion from the Morgan–Russell scale [51]. These studies also included menstrual recovery as part of their weight maintenance definition. This dilutes the definition of weight maintenance, which cannot be used across wider samples, including men.

Furthermore, most studies took one measure of weight at follow-up and used a weight cut off to establish whether participants had maintained their weight throughout this time period, instead of taking multiple measurements to ascertain sustained weight maintenance. This approach does not necessarily represent a true measure of ‘maintenance’, as it is possible that participants may have lost weight in between follow-up measures.

Taken together, there is a lack of consensus between researchers in the definition of weight maintenance, as well as a need for more robust and consistent measurement methods. We hope this paper stimulates the debate. It is important to improve this before trying to explore more complex concepts, such as recovery.

### Strengths and limits

This study gives voice to the lack of clarity around the concept of eating disorders recovery, alongside the impact that this could have on treatment. To our knowledge this is the first systematic review on papers looking at factors affecting weight maintenance.

The present study has several limitations. Findings should be treated with caution given the small number of available studies, as well as the heterogeneity in their design, intervention and follow-up durations. The differences make it difficult to make comparisons between studies and find patterns in results, highlighting the need for a common definition of weight maintenance across studies [45]. We included studies that included menstruation as part of their criteria. It is possible that this may have skewed findings, for having an additional criterion for maintenance may reduce the likelihood that certain predictors are found significant, or alternatively, other factors may hold more importance. In addition, most studies had primarily white female samples, particularly so in the studies that included menstruation resumption as part of their criteria. Men and non-white samples are more likely to have poorer outcomes [44], therefore significant predictors identified in this review may not apply to those populations.

### Implications

Future research must focus on developing a clear concept of weight maintenance as it pertains to the eating disorders and particularly AN. Research and common clinical observation suggests that weight maintenance is the first step to full psychological recovery [19]. In addition, there lacks a clear consensus on the definitions of recovery and relapse, and better understanding weight maintenance may help contribute to rectifying this. Understanding the factors that predict weight maintenance can help clinicians adapt existing treatments to focus on targeting these factors, with the aim of supporting patients to maintain their weight after treatment and work towards full recovery.

Avenues that may be explored by future research include replicating studies looking at BMI throughout treatment, in order to increase reliability in the findings around weight variables. Future research should also investigate further the relationship between body image and long-term weight maintenance, given this review’s mixed findings.

## Conclusion

The present study aimed to scope the literature on the factors predicting weight maintenance after acknowledging that this is a critical factor for recovery, and the inconsistent findings and definitions of recovery.

The current literature on weight maintenance suggests that a higher BMI at admission and discharge are the strongest predictors of long-term weight maintenance. Mixed findings have been found for biological and psychological factors. It is important for readers to interpret these findings with care, and to combine this with a wider understanding of what is important for AN patients, rather than using these results in isolation to promote a purely medical model of recovery. The findings provide important implications for future research as they highlight the need for a common definition of weight maintenance, as well as the need to compare differences between adult and adolescent samples so we can ensure that treatments are tailored to their individual needs. Further research should aim to develop a clear definition of weight maintenance and investigate predictive factors, including how BMI and weight gain processes account for weight maintenance, and elucidate the role of psychological processes in weight maintenance.

### What is already known on this subject?

There exists extensive research on eating disorder recovery, but there are different views on how this should be defined and measured. Several factors have been suggested to predict long-term recovery, yet the recovery landscape remains unclear due to the lack of consensus on the definition of recovery and on the factors deemed to predict recovery.

### What this study adds?

This study adds an understanding of how weight maintenance is conceptualised in eating disorder research and an initial understanding of factors predicting weight maintenance, upon which future research can build.

## Data Availability

The datasets used in this study are available from the corresponding author on reasonable request.
